# Is Autologous Fecal Microbiota Transfer after Exclusive Enteral Nutrition in Pediatric Crohn’s Disease Patients Rational and Feasible? Data from a Feasibility Test

**DOI:** 10.3390/nu15071742

**Published:** 2023-04-02

**Authors:** Hannes Hoelz, Jeannine Heetmeyer, Anastasia Tsakmaklis, Andreas Hiergeist, Kolja Siebert, Federica De Zen, Deborah Häcker, Amira Metwaly, Klaus Neuhaus, André Gessner, Maria J. G. T. Vehreschild, Dirk Haller, Tobias Schwerd

**Affiliations:** 1 Department of Pediatrics, Dr. von Hauner Children’s Hospital, University Hospital, LMU Munich, 80337 Munich, Germany; 2 Clinical Microbiome Research Group, Department of Internal Medicine I, University Hospital of Cologne, 50931 Cologne, Germany; 3 Institute for Microbiology and Hygiene, University Hospital Regensburg, 93053 Regensburg, Germany; 4 Chair of Nutrition and Immunology, Technical University Munich, 85354 Freising-Weihenstephan, Germany; 5 ZIEL-Institute for Food and Health, Technical University Munich, 85354 Freising-Weihenstephan, Germany; 6 Section of Infectious Diseases, Department of Internal Medicine II, University Hospital Frankfurt, Goethe University Frankfurt, 60596 Frankfurt am Main, Germany

**Keywords:** pediatric IBD, Crohn’s disease, fecal microbiota transfer, autologous FMT, exclusive enteral nutrition

## Abstract

Background: Exclusive enteral nutrition (EEN) is a highly effective therapy for remission induction in pediatric Crohn’s disease (CD), but relapse rates after return to a regular diet are high. Autologous fecal microbiota transfer (FMT) using stool collected during EEN-induced clinical remission might represent a novel approach to maintaining the benefits of EEN. Methods: Pediatric CD patients provided fecal material at home, which was shipped at 4 °C to an FMT laboratory for FMT capsule generation and extensive pathogen safety screening. The microbial community composition of samples taken before and after shipment and after encapsulation was characterized using 16S rRNA amplicon sequencing. Results: Seven pediatric patients provided fecal material for nine test runs after at least three weeks of nutritional therapy. FMT capsules were successfully generated in 6/8 deliveries, but stool weight and consistency varied widely. Transport and processing of fecal material into FMT capsules did not fundamentally change microbial composition, but microbial richness was <30 genera in 3/9 samples. Stool safety screening was positive for potential pathogens or drug resistance genes in 8/9 test runs. Conclusions: A high pathogen burden, low-diversity microbiota, and practical deficiencies of EEN-conditioned fecal material might render autologous capsule-FMT an unsuitable approach as maintenance therapy for pediatric CD patients.

## 1. Introduction

Fecal microbiota transfer (FMT) from a healthy donor is an established treatment for recurrent *Clostridioides difficile* infection, with remarkable clinical efficacy and durable engraftment of donor strains [1,2,3]. Currently, FMT is being investigated as a promising treatment modality for other diseases associated with intestinal dysbiosis, including inflammatory bowel disease (IBD). While healthy donor FMT has been shown to effectively induce clinical remission and mucosal healing in ulcerative colitis (UC) patients [4,5,6,7], recent studies similarly suggest a beneficial effect in Crohn’s disease (CD) patients [8,9]. In CD, higher engraftment of donor strains has been linked to prolonged maintenance of remission and improved clinical outcomes [10,11]. However, a variety of donor and recipient characteristics have been shown to affect the efficacy of FMT in IBD, making the selection of a suitable donor challenging [4,10,12,13,14]. Previous studies and meta-analyses show a good safety profile of FMT [1,15,16], but there is still a potential risk of disease relapse in patients with IBD [17]. While the risk of pathogen transmission can be minimized but not eliminated by rigorous donor screening and quarantine [18,19,20], additional concerns about the long-term effects of FMT have been expressed, including the transfer of a donor microbiota suspected to have pro-carcinogenic properties or to predispose to metabolic disorders like obesity [21,22,23,24].

To address these limitations of “allogenic” FMT, which are particularly relevant in vulnerable patient groups such as children, autologous FMT has been proposed as an alternative approach in which the recipient’s own fecal material is collected during a healthy state and later used to maintain or restore it [13,25]. As proof of principle, in a recent randomized controlled trial of autologous FMT in obese patients, encapsulated stool collected during a period of diet-induced weight loss and administered during the expected weight regain phase was found to preserve weight loss and reduce insulin rebound, but only in participants from the green Mediterranean diet group. This diet based on reduced meat consumption and increased intake of fish, green tea, and Mankai duckweed led to a significant shift in microbial composition and metabolic pathways [26]. Together with results from previous dietary studies [27,28], these findings suggest that, similarly to IBD, dietary measures could effectively shape the microbiota toward an “optimized” state for subsequent autologous FMT.

In CD, one such dietary intervention might be exclusive enteral nutrition (EEN), which involves the exclusive administration of a liquid polymeric or elemental formula for a period of 6–8 weeks. In addition to its high clinical efficacy, favorable safety profile, and additional benefits, including improvement of nutritional status and growth, EEN achieves significantly higher rates of mucosal healing compared to oral corticosteroids and is therefore recommended as first-line therapy for induction of remission in pediatric CD patients [29,30,31,32,33,34]. The beneficial effects of EEN are accompanied by a substantial shift in gut microbiota and metabolome composition, with, however, significant interindividual variability [29,35,36,37,38,39]. Despite all its advantages, EEN is not a long-term treatment option, and both microbial changes and clinical improvement are not preserved upon reintroduction of a regular diet, with 42–67% of patients relapsing within one year of EEN cessation [33,40,41,42]. Based on the hypothesis that EEN-induced changes in microbial composition and function significantly contribute to treatment efficacy, we hypothesized that EEN-conditioned microbiota could be used in an autologous capsule FMT approach to maintain remission in pediatric CD patients.

To ensure that FMT products are consistently produced and quality controlled, donor feces collection and preparation for FMT should follow a standard protocol [43]. In addition, and according to current regulation for FMT in humans, fecal material must meet certain quality criteria, such as the Bristol Index of 3–4, an amount of fecal material that allows infectious disease screening and processing, and the absence of pathogens or antibiotic-resistance genes. Furthermore, a high level of alpha diversity in the donor material is required (Figure S1). For allogeneic FMT, either on-site stool donation is propagated or donors should hand in their feces within 2 h after defecation to avoid environmental contamination and to ensure microbial stability [43,44]. In the case of autologous FMT, stool donation poses a logistical challenge depending on the location of the treating hospital and the FMT laboratory. Therefore, we aimed to evaluate the feasibility of at-home stool collection during EEN-induced remission and of further processing fecal material into FMT capsules, including the evaluation of quality and quantity of stool donations, analysis of microbial composition, and a complete safety screening for pathogens.

## 2. Materials and Methods

### 2.1. Eligibility and Recruitment

The study was approved by the local LMU ethics committee (approval no. 17-801, approved 18 March 2018) and registered at the German Clinical Trials Register (accession no. DRKS00013306, registered 19 March 2018). Pediatric patients aged 3–17 years with active CD requiring induction therapy with EEN according to consensus guidelines were recruited in the Munich pediatric IBD cohort study [34]. This monocentric observational trial aims at investigating the functional relevance of gut microbial composition and function for immune regulation and disease progression in childhood-onset IBD. Written parental or guardian informed consent was obtained for all participants, who themselves provided informed assent. Participants, parents, or guardians received no financial compensation or gifts.

### 2.2. Study Design

For EEN induction treatment, study participants used Modulen^®^ IBD (Nestlé Health Science, Frankfurt/Main, Germany) as an exclusive source of nutrition for 6–8 weeks, as described in Frivolt et al. [40]. For assessment of clinical disease activity, the weighted pediatric Crohn’s disease activity index (wPCDAI) was determined at baseline, once between weeks 2 and 4 of EEN, once between weeks 5 and 8 of EEN, and once between weeks 1 and 2 after completion of EEN [45]. If possible, fecal calprotectin levels were obtained in similar time intervals as the wPCDAI; otherwise, they were determined once at baseline, once during, and once after EEN. Study participants were asked to donate stool at home during EEN in a provided Fecotainer (DaklaPack^®^ Europe, Lelystad, The Netherlands). After immediate transport to the hospital at 4 °C, Fecotainers were packed and shipped overnight at 4 °C to the GMP-certified FMT laboratories in Cologne or Regensburg. Temperature stability was recorded during the entire transport.

On arrival at the FMT laboratory, processing and quality control were performed according to the European consensus conference on FMT in clinical practice and the consensus report of the United European Gastroenterology (UEG) FMT working group (see Figure S1, showing a flow chart of the FMT capsule manufacturing process). Initially, stool donations were inspected for macroscopic impurities (i.e., no blood, mucus, or urine) and analyzed for fecal mass (at least 65 g) and stool consistency (Bristol stool scale 3–4) (see Figure S1). A comprehensive FMT donor safety screening for pathogens was performed (see Table S1), and samples for quality control were taken. For encapsulation, stool samples were homogenized with saline (0.9% NaCl), filtered, and centrifuged to remove larger components such as food particles. After centrifugation of the supernatant and resuspension of the pellets in saline and glycerol, stool suspensions were aliquoted into capsules, which were then frozen at −80 °C. To compare microbial composition pre-transport, post-transport, and post-encapsulation, three samples were transferred into tubes containing DNA stabilizer (Invitek Molecular, Berlin, Germany) (one stool sample taken at home immediately after defecation, one sample taken from the Fecotainer after arrival at the FMT laboratory, and one sample of the content of one frozen capsule) and subsequently analyzed via 16S rRNA amplicon sequencing.

As high alpha diversity represents an important quality criterion for FMT products, a Shannon index > 2 and a microbial richness > 30 genera have been defined as prerequisites for FMT capsule production by the FMT laboratory in Cologne. These definitions are based on the following thoughts: the alpha-diversity can be expressed by a wide range of indices, which are all calculated by different formulas taking different aspects into account; the Shannon index favors an even distribution. Hence, the same number of taxa may result in different scores. A microbiome with one dominant taxon would yield a lower Shannon index than a microbiome with an evenly distributed composition. On the downside, an optimal (even) distribution of taxa might result in a high Shannon index despite a low number of taxa. Therefore, the FMT laboratory in Cologne also uses richness, i.e., the number of taxa within a sample, as a secondary indicator for diversity.

### 2.3. 16S rRNA Sequencing

Samples were prepared for sequencing at the sequencing core facilities in Freising (Core Facility Microbiome; ZIEL—Institute for Food & Health) and Regensburg (Institute for Clinical Microbiology and Hygiene, Core Facility Microbiome). Microbial DNA was isolated from about 150 mg of each fecal sample by bead beating (see [46]), followed by purification using guanidinium thiocyanate and N-lauroylsarcosine to remove cellular components and polyvinylpyrrolidone to remove phenolics, as well as cleaning of the DNA with RNase A and the NucleoSpin gDNA Clean-up Kit (Machery-Nagel, Dueren, Germany) (Freising core facility). In Regensburg, microbial DNA was isolated by bead beating on a TissueLyzer II instrument (Qiagen, Hilden, Germany), followed by purification of stool lysates by the MagNA Pure 96 system (Roche, Pernzberg, Germany) (Regensburg core facility). Microbiome sequencing was conducted in Regensburg with a DIN EN ISO 15189-accredited workflow. Briefly, the V1–V3 variable regions of the 16S rRNA gene were amplified in each sample using universal primers S-D-Bact-0008-c-S-20 and S-D-Bact-0517-a-A-18, and the resulting amplicons were sequenced on an Ion GeneStudio S5 Plus instrument (Thermo Fisher Scientific, Germering, Germany). Raw sequencing data were retrieved from TorrentSuite 5.18 and further subjected to Cutadapt 4.1 for adapter and primer removal, demultiplexing, and Trimmomatic 0.4 for sliding-window-based quality filtering [47,48]. Bacterial 16S rRNA copy numbers were quantified from extracted DNA using a 16S qRT-PCR as described before [49]. Absolute bacterial biomass was calculated using the amount of bacterial 16S rRNA copy numbers in 1 g of stool, taking into account the initial stool weight and dilutions during processing.

### 2.4. Analysis of Bacterial Composition

Sequencing data were preprocessed using a VSEARCH 2.21.1-based pipeline [50]. Reads with an expected error rate above 5 were removed. Zero-radius OTUs (zOTUs) were built from quality-filtered reads applying an alpha value of 2 and a minimum size of 5 reads. Chimeric sequences were removed using the uchime3_denovo algorithm. Filtered reads with 98 percent pairwise identities were mapped back to non-chimeric zOTUs with the usearch_global algorithm. Taxonomy was assigned in R version 4.1.3 using the IDTAXA algorithm of DECIPHER 2.22 and the All-Species Living Tree Project database version 01.2022 [51,52].

### 2.5. Calculation of Diversity Indices

Diversity analysis was performed with mia using the default parameters [53]. Species richness (zOTUs), Shannon index, and Shannon effective (number of species) were calculated to assess alpha diversity. Beta diversity was assessed by unweighted and weighted UniFrac distances. A Principal Coordinates Analysis (PCoA) plot based on unweighted and weighted UniFrac matrices was constructed to demonstrate the overall dissimilarity of bacterial communities between study participants. A heatmap of the 40 most abundant genera was generated with pheatmap 1.0.12 after centered log and Z-transformation of zOTU counts. Features and samples were hierarchically clustered by the complete linkage method.

### 2.6. Statistical Analysis

The statistical significance of changes in wPCDAI and fecal calprotectin levels was assessed by a one-way ANOVA with Tukey´s multiple comparisons test. Differences between groups of effective Shannon diversity were tested for significance using the Wilcoxon signed-rank test. A PERMANOVA analysis was used to evaluate the significance between groups of the Bray–Curtis, unweighted, weighted, and generalized UniFrac distances. Linear models were fitted to distance matrices using the adonis2 command in the vegan package [54]. Patients were included as covariates in the model to control for individual differences. Results were considered significant at *p* ≤ 0.05. For pairwise comparisons, *p*-values were adjusted by the false discovery rate using the Benjamini–Hochberg procedure.

A TSS normalized, log-transformed linear model was used to identify significantly different zOTUs before and after shipment, as well as after shipment and after encapsulation [55]. The timepoint of sampling was included as a fixed effect and the individual patients as random effects in the model. Correction for multiple testing was performed using the Benjamini–Hochberg FDR threshold of 0.25. A cutoff value of 0.05 was applied for the uncorrected *p*-value to plot the relative abundances of each group.

## 3. Results

### 3.1. Study Population

**Figure 1:**
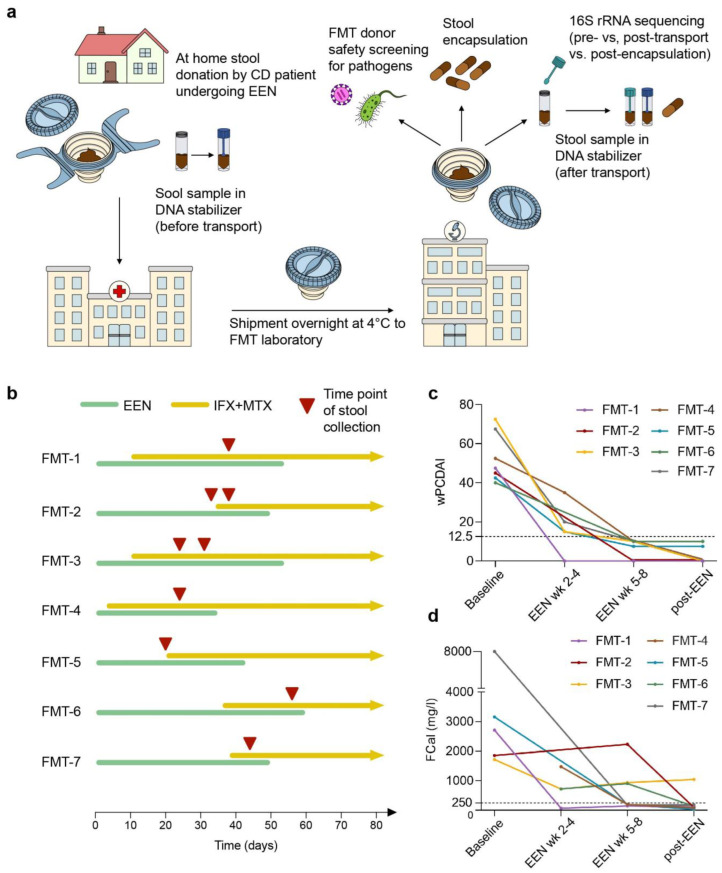
Clinical course of study participants and time point of stool donation. (**a**) Study design. Study participants were asked to donate stool at home during EEN treatment. After immediate transport to the clinic at 4 °C, Fecotainers were packed and shipped overnight at 4 °C to the GMP-certified FMT laboratory. Stool donations were then analyzed for fecal mass and stool consistency; a comprehensive FMT donor safety screening for pathogens was performed; and FMT capsules were produced. (**b**) Illustration of treatment periods and time points of stool donation for FMT capsule production. Green: induction therapy with EEN. Ochre: maintenance therapy with IFX and MTX. Red triangle: time point of stool collection. (**c**) Weighted PCDAI (wPCDAI) scores of study participants at baseline, at weeks 2–4 of EEN (EEN week 2–4), at weeks 5–8 of EEN (EEN week 5–8), and 1–2 weeks after completion of EEN (post-EEN). (**d**) Fecal calprotectin (FCal) concentrations of study participants at baseline, at weeks 2–4 of EEN (EEN week 2–4), at weeks 5–8 of EEN (EEN week 5–8), and 1–2 weeks after completion of EEN (post-EEN). Test for significance was performed using a one-way ANOVA with Tukey´s multiple comparisons test (confidence level of 95%; definition of statistical significance: *p* < 0.05). Abb.: CD = Crohn’s disease; EEN = exclusive enteral nutrition; FCal = fecal calprotectin; FMT = fecal microbiota transfer; IFX = infliximab; MTX = methotrexate; wk = week; wPCDAI = weighted pediatric Crohn’s disease activity index.

To assess the general feasibility of an autologous FMT approach in pediatric CD patients, we set up test runs of at-home stool donation, refrigerated shipment to a certified FMT laboratory, and local processing into FMT capsules (Figure 1a). Seven pediatric CD patients with a mean age of 13.8 ± 2.1 years provided fecal material (designated FMT-1 to FMT-7).

The demographic and clinical characteristics of the seven patients (4/7 female) are listed in Table 1. Five of seven patients were newly diagnosed, while patients FMT-3 and FMT-4 experienced a disease relapse requiring induction therapy with EEN. The mean age at diagnosis was 12.4 ± 3.2 years. Most patients had ileocolonic (3/7) or colonic (2/7) disease. One patient had terminal ileitis and another patient had isolated small bowel disease. Additional upper gastrointestinal involvement was found in all patients. While disease behavior mostly corresponded to non-stricturing, non-penetrating disease, intra-abdominal fistulas could not be ruled out in patient FMT-5, whose MRI showed extensive adherence of small bowel loops. This patient also presented with an intersphincteric fistula and recurrent perianal and perirectal abscesses. In the other patient with perianal disease (FMT-3), mild anorectal stenosis, multiple anal fissures, and a small perianal fistula were observed. Linear growth impairment according to the Paris classification was found in three patients from our cohort [56]. One patient presented with severe malnourishment according to the WHO definition of a BMI z-score <−2 (see Table S2). Clinical disease activity ranged from mild to severe (Table 1).

After the diagnostic work-up, all patients received EEN for six–eight weeks. Due to the presence of predictors of poor outcome [57], all seven patients were started on infliximab (IFX) with methotrexate (MTX) as co-medication in parallel to EEN to prevent development of anti-drug antibodies. Treatments and time points of at-home stool donation are illustrated in Figure 1b and in Figure S2. Two patients (FMT-2 and FMT-3) provided samples for two test runs. Previously, we observed that fecal bacterial communities were significantly altered after 2 weeks of EEN [36]. All patients had completed at least three full weeks of EEN at the time of stool donation, and most samples, except for two, were taken after initiation of the maintenance therapy with infliximab and methotrexate.

Induction treatment with EEN led to a rapid and significant drop in symptoms as well as wPCDAI scores (*p* < 0.001 for pre-EEN vs. EEN week 2–4, *p* < 0.001 for pre-EEN vs. EEN week 5–8, see Figure 1c), and improvement of BMI z-scores (*p* = 0.069 and Table S2). All study participants achieved clinical remission by the end of EEN. A significant reduction of fecal calprotectin levels was also observed upon treatment (*p* = 0.026 for pre-EEN vs. EEN week 2–4, *p* = 0.008 for pre-EEN vs. EEN wk 5–8, see Figure 1d), indicating improvement of intestinal inflammation. Except for one patient (FMT-3) with ileocolitis and severe clinical and endoscopic activity, fecal calprotectin levels dropped below 250 mg/L in all patients following EEN and on maintenance therapy with infliximab and methotrexate (Figure 1d). At the time of the stool donation, six of seven patients were in clinical remission, but clinical remission together with low fecal calprotectin was only observed in patient FMT-7 (see Figure S2).

### 3.2. FMT Capsule Production Is Limited by Quantitative and Qualitative Deficits of Fecal Material

The results from the nine test runs of at-home stool donation, transport to the FMT laboratory, and processing into FMT capsules are summarized in Table 2. All patients had completed at least three full weeks of EEN by the time of stool donation. Weight of stool donations varied between 21 and 240 g, excluding one case in which stool leaked from the Fecotainer during transport. The minimum weight of 65 g required for production of a full batch of FMT capsules was exceeded in five of the nine samples (Table 2).

We further observed major differences in stool consistency under EEN. While three patients had semi- to fully liquid stools, solid lumps were found in two patients. Only the two samples from patients FMT-6 and FMT-7 were of adequate consistency (Bristol stool scale 3–4) for FMT capsule production according to manufacturing standards. In all test runs, the maximum possible number of stool capsules was produced, irrespective of the usual specifications of the FMT laboratory (see Figure S1). A full batch of 30 capsules was produced for five patients (Table 2).

**Figure 2:**
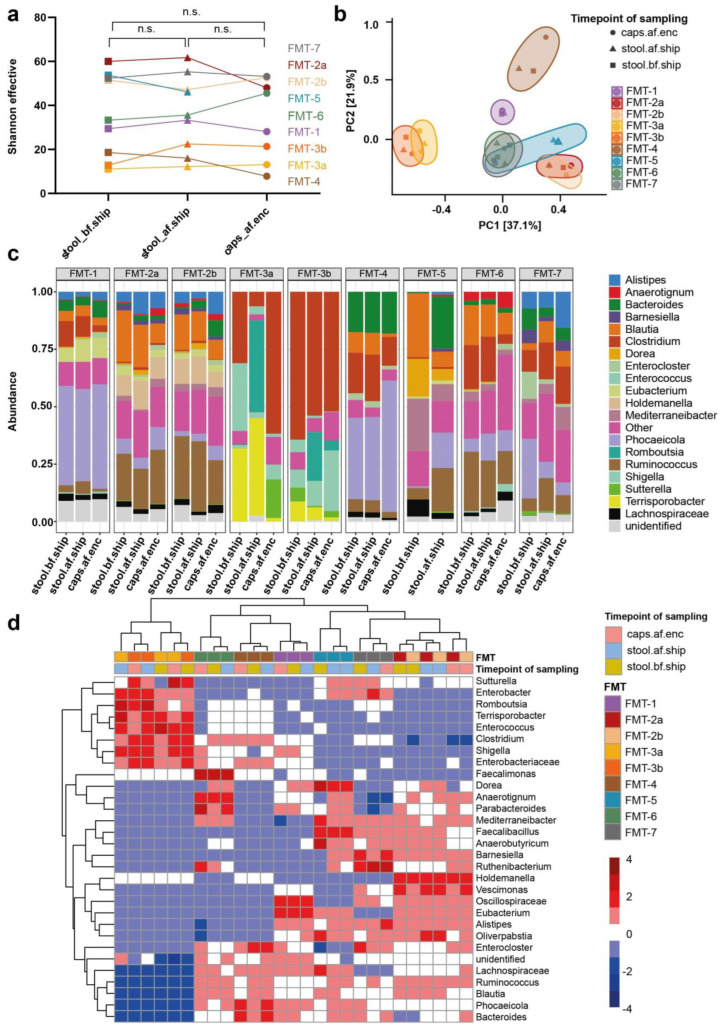
Microbial composition analysis of fecal samples pre- and post-shipment and after encapsulation as determined by 16S rRNA sequencing. (**a**) Shannon’s effective number of species plotted for fecal samples pre- (■), post-shipment (▲), and after stool encapsulation (●). The test for significance was calculated using a paired t-test (confidence level: 95%, definition of statistical significance: *p* < 0.05); n.s. = not significant. (**b**) Beta diversity measured by weighted UniFrac dissimilarity and principal coordinates analysis (PCoA) plotted for fecal samples pre- (■) post-shipment (▲), and after encapsulation (●). A PERMANOVA analysis was used to evaluate the significance between the groups. A pairwise comparison of stool before and after shipment (*p* = 0.41) and stool before shipment and after encapsulation (*p* = 0.07) showed no statistically significant difference, respectively. The comparison of stool after shipment and after encapsulation (*p* = 0.04) showed a statistically significant difference. (**c**) Stacked bar charts of the relative abundance of the top 20 bacterial genera pre- (stool.bf.ship), post-shipment (stool.af.ship), and after encapsulation (caps.af.enc). (**d**) A heatmap showing the relative abundances of the top 40 bacterial genera in stool samples from study participants pre- (stool.bf.ship, khaki green), post-shipment (stool.af.ship, light blue), and after encapsulation (caps.af.enc, light red), as determined by 16S rRNA sequencing. A distance tree based on hierarchical clustering illustrates the relationship between the respective samples and genera. Note: Patients FMT-2 and FMT-3 provided fecal material for two test runs (FMT-2a/b and FMT-3a/b). Two samples taken post-transport were analyzed for patient FMT-5, and no capsules could be produced from the fecal material donated by this patient due to stool leakage from the Fecotainer during transport.

We further analyzed the stool samples by 16S rRNA gene sequencing to determine microbial composition and diversity (see Figure 2). A high level of alpha diversity is associated with a healthy microbiota and successful allogeneic FMT in UC studies [14]. As a prerequisite for allogeneic FMT, a Shannon index >2 and a microbial richness >30 genera are required (see Figure S1). The bacterial richness under EEN therapy varied widely between patients and ranged between 15 and 49 zOTUs at the genera level (Table 2). Only in four patients (five samples), fecal donations were characterized by high microbial richness, with more than 30 genera detected. In one patient (FMT-6), bacterial richness was borderline, while particularly low numbers of genera were found in the remaining two patients (see Table 2 and Figure S3b, showing that two patients (FMT-3 and FMT-4) had <20 observed genera).

### 3.3. Transport and Processing of Stool Donations into FMT Capsules Induce Minor Changes in Microbial Composition

To evaluate whether at-home stool collection, cooled transport at 4 °C, and encapsulation affect microbial composition, stool samples taken at the time of defecation and after transport to the laboratory, as well as the content of a frozen FMT capsule from the respective patient, were analyzed by 16S rRNA gene sequencing. Both alpha- and beta-diversity were not significantly affected by cooled transport as evidenced by stable Shannon effective (*p* = 0.65 for stool pre- vs. post-shipment) and dense clustering of pre- and post-shipment samples from one patient in an unweighted and weighted UniFrac (see Figure 2a,b, Figures S3 and S4). Pairwise comparison based on Bray–Curtis, unweighted and weighted, as well as generalized UniFrac using PERMANOVA analysis with correction for patients as covariates, showed no significant difference for stool pre- vs. post-shipment (see Table S3, showing the *p*-values of pairwise comparison for stool pre- vs. post-shipment: Bray–Curtis: *p* = 0.10; unweighted UniFrac: *p* = 0.12; weighted UniFrac: *p* = 0.41; and generalized UniFrac: *p* = 0.09). Stool processing with encapsulation resulted in stable alpha-diversity represented by Shannon effective (*p* = 0.53 for stool post-shipment vs. encapsulated stool and *p* = 0.98 for stool pre-shipment vs. encapsulated stool), while beta-diversity with the exception of the unweighted UniFrac distance, significantly changed for the pairwise comparison of stool post-shipment vs. encapsulated stool (see Table S3, showing the following significant *p*-values of the pairwise comparison for stool post-shipment vs. encapsulated stool: Bray–Curtis: *p* = 0.03; weighted UniFrac: *p* = 0.04; and generalized UniFrac: *p* = 0.02).

Analyses of relative abundance at the genus level revealed overall stability of relative microbial composition before and after shipment of stool samples (see Figure 2c,d and Figure S5), except for some shifts most apparent in two patients. In the first sample of patient FMT-3 (FMT-3a), a relatively large fraction of *Enterococcus* was found, which was significantly less in the post-shipment sample, while the relative abundance of *Romboutsia* strongly increased. Microbial changes at the genus level could also be observed in the stool samples from patient FMT-5 (Figure 2c). Though not statistically significant, differences in microbial composition between post-shipment and encapsulated stool were mainly explained by reduced relative abundances of three zOTUs from the *Dorea* genus and two zOTUs from the *Ruminococcus* genus after stool encapsulation. In contrast, a statistically non-significant enrichment in the relative abundance of individual zOTUs from *Anaerotruncus*, *Clostridium,* and unclassified *Enterobacteriaceae* after stool encapsulation was observed (see Figure S6, showing no statistically significant differences in differential abundance of zOTUs).

Analysis of 16S rDNA copies by qRT-PCR revealed increased absolute bacterial counts in four samples after shipment at 4 °C compared to bacterial counts pre-transport (FMT-2b, FMT-3b, FMT-4, and FMT-5). Following processing and encapsulation of stool samples, bacterial content in the capsules was much lower than in the original stool samples in all patients except FMT-3 (see Figure S7). Additionally, after normalization, all zOTUs were screened for significant changes in absolute abundances pre- and post-shipment. Though differences in normalized zOTU levels were observed, these changes were not found to be significant (see Figure S8).

### 3.4. Safety Screening Reveals High Prevalence of Pathogen Colonization

**Figure 3:**
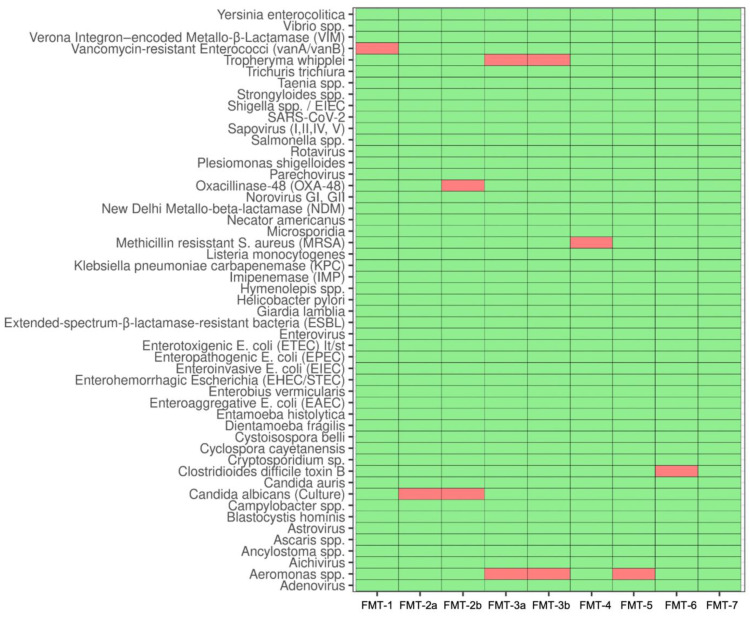
Results from infectious disease safety screening. In-depth FMT donor screening was performed on stool samples from the seven study participants. Red boxes indicate detection of the respective pathogen. Patients FMT-2 and FMT-3 provided fecal material for two test runs (FMT2a/b and FMT-3a/b).

As part of the FMT capsule production, we performed an in-depth pathogen screening according to allogeneic healthy-donor FMT requirements (Figure 3). We detected pathogens and drug-resistant strains (or their genes) in all stool samples except for one (from patient FMT-7), including Toxin-B from *Clostridioides difficile*. In patient FMT-2, the antibiotic resistance gene oxacillinase-48 was detected. Strikingly, we also detected *Tropheryma whipplei* via PCR in both samples from patient FMT-3. *Aeromonas species*, which are tolerated in healthy-donor FMT, were found in two study participants.

### 3.5. Stool Donations from Pediatric CD Patients under EEN Are Not Suitable for Autologous FMT Capsule Production

**Figure 4:**
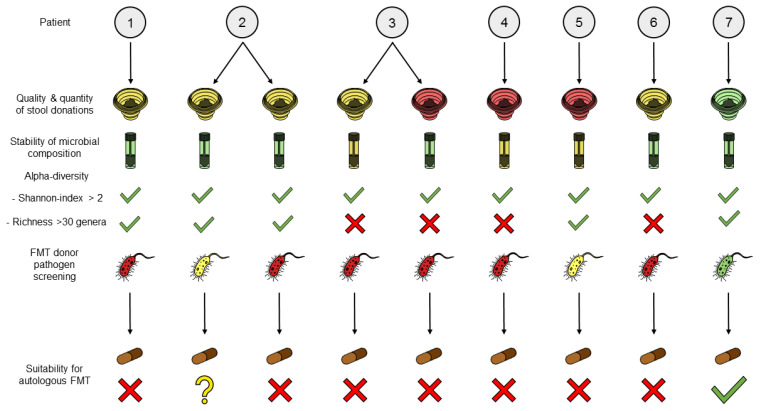
Suitability of stool donations from pediatric CD patients for autologous FMT. Numbers from 1 to 7 represent patients FMT-1 to FMT-7. Illustration of quality and quantity of stool donations (Fecotainer: green: standard requirements of FMT laboratory for FMT capsule production met, including fecal biomass >65 g, Bristol stool scale 3–4, and high alpha diversity, represented by Shannon index >2 and a richness above 30 genera, yellow: deviations of fecal weight and/or consistency and/or microbial richness, red: fecal weight and/or consistency insufficient resulting in failure to produce full batch of FMT capsules), stability of microbial composition during transport (Tube: green: comparable microbial composition and absolute abundance pre- and post-transport, yellow: increased absolute abundance of bacteria and/or significant changes in relative microbial composition at the genus level post-transport) and results from FMT donor pathogen screening (Bacterium: green: no pathogens detected, yellow: tolerable pathogens detected, and red: pathogens detected) for the nine FMT capsule production test runs.

Based on the previously described practical and safety aspects, we then evaluated the suitability of the stool donations for autologous FMT (Figure 4). We identified major deficiencies in both quantity and quality of most stool donations, including an insufficient amount of fecal material, inadequate stool consistency (Bristol stool scale < 3 or > 4) and/or low microbial diversity (with less than 30 genera found in four samples from three patients). Transport at 4 °C did not significantly alter microbial composition, but some changes could be observed at the genus level in the fecal material of patients FMT-3 and FMT-5 in particular, and the absolute abundance of bacteria notably increased in two stool samples (see Figure S7). In four patients, we detected pathogens considered an absolute contraindication to FMT. In patient FMT-2, both the solid consistency of the stool and the detection of an antibiotic resistance gene in the second sample limited its suitability for autologous FMT. Patient FMT-5 could not be fully evaluated due to damage to the Fecotainer during transport, resulting in failure to produce any FMT capsules, but the semi-liquid consistency of the stool did not correspond to standard requirements for FMT capsule production either way. Only in patient FMT-7 was the stool donation of sufficient quantity and quality, and the pathogen screening was negative, making it suitable for a potential autologous FMT.

## 4. Discussion

In this pilot study, we aimed to assess the general feasibility of an autologous FMT approach in pediatric CD patients by evaluating the suitability of at-home stool donations collected during EEN for the production of FMT capsules. We found that the microbial composition of fresh stool samples as assessed by 16S rRNA gene sequencing was not significantly altered by shipment at 4 °C or encapsulation, supporting the potential of at-home donation as an alternative to on-site sampling and indicating a high biological quality of frozen FMT capsules. However, these findings are based on a very small cohort of seven patients, and additional tests, including bacterial viability assays or culturing, would be necessary to confirm the presence and conservation of live bacteria in stool samples following transport and processing. Still, we observed some alterations of microbial composition and DNA content, notably an increase in the absolute abundance of organisms post-shipment in some samples. The effects of such changes, potentially caused by “uncontrolled” bacterial growth, on long-term microbial composition and function and, therefore, the quality of the FMT product, remain unclear. Furthermore, the Fecotainer system does not allow anaerobic sampling, possibly leading to a reduction in potentially beneficial obligate anaerobic microorganisms such as bacteria from the *Clostridiales* order [58,59]. These limitations could be addressed by optimizing shipping procedures, but scheduled same-day delivery is likely not feasible given the variability of bowel habits, particularly under EEN treatment. In any case, current regulations require on-site stool donations as a mandatory step in the manufacturing process of FMT capsules. A modified study design involving on-site sampling would, however, pose a logistic challenge to patients and their families.

In addition, we observed that most stool donations did not fulfill the required quality criteria, including a sufficient quantity and adequate consistency. As the formula does not contain any fibers, stool irregularities and diarrhea in particular are common side effects of EEN treatment [60]. Furthermore, we cannot exclude that some patients used a higher concentration of Modulen^®^ IBD (Nestlé Health Science) formula to improve palatability, which might have had an effect on water secretion/absorption in the intestine due to increased osmolality. In our cohort, a full batch of FMT capsules could not be produced in three of the nine test runs due to problems with the fecal material, including stool leakage from the Fecotainer in one case. In addition to these practical issues the detection of pathogens in the majority of stool samples was found to be a major limitation of the autologous FMT approach. A variety of changes in both composition and function of the gut microbiota have been described in CD patients, including a reduction in biodiversity, a loss of health-associated species, and an alteration of metabolic profiles [61,62,63,64]. This state of imbalance, referred to as intestinal dysbiosis, not only contributes to the abnormal activation of the mucosal immune system [65], but also leads to a loss of pathogen colonization resistance and thereby to a high prevalence of both asymptomatic colonization and symptomatic infection with potentially pathogenic organisms [66,67,68,69,70,71]. In line with these observations, we detected toxin-bearing *C. difficile* or drug-resistant bacteria in more than half of our study population, excluding the use of the respective fecal material for an autologous FMT approach.

Interestingly, we also detected *Tropheryma whipplei* in one patient, commonly known as the causative organism of Whipple’s disease, a rare and potentially fatal multisystem disorder [72]. Studies have found varying prevalences of chronic asymptomatic carriage in different populations [73,74]. While such colonization is likely the case in our patient, *Tropheryma whipplei* has also been linked to acute gastroenteritis in young children [75]. Few case reports suggest that *Tropheryma whipplei* infection can mimic CD [76,77,78], but a general association with IBD or an increased carrier frequency in CD patients have not been reported.

Overall, as the production of microbiologically safe FMT capsules was successful in only one case, we concluded that autologous FMT using EEN-conditioned fecal material may not be feasible for a substantial number of pediatric CD patients. Other studies investigating allogenic FMT for maintenance of remission in CD or autologous FMT for the prevention of antibiotic-resistant bacterial infections and treatment of graft-versus-host disease have used alternative delivery modes, including administration of fecal suspensions via rectal enema or endoscopy, thereby avoiding practical issues of FMT capsule production [10,79,80]. However, in addition to being more invasive and therefore less suitable for a pediatric study population, implementation of alternative delivery routes would not solve the problem of high pathogen carrier rates seen in our cohort.

To our knowledge, this is the first study to explore the feasibility of autologous FMT in pediatric CD patients. Despite the limited number of participants, our study population was relatively heterogeneous in terms of age, gender, and disease classification and thereby fairly representative of pediatric CD, suggesting that the previously described limitations of the autologous FMT approach were not specific to a particular demographic or disease phenotype. While the primary goal of our study was to assess the general feasibility of autologous FMT, including practical and safety-related requirements, we did not evaluate whether maintenance therapy with FMT capsules itself was feasible. Even in cases of successful stool encapsulation and exclusion of pathogens, to transfer a biologically effective dose of microbiota, ingestion of a relatively large number of capsules is likely necessary, which might be challenging in young patients due to capsule size and difficulties swallowing. Furthermore, the time period of EEN-induced remission is limited, which further complicates the manufacturing process of a sufficient amount of individualized FMT capsules.

Regardless of these feasibility issues, the rationale for autologous FMT following EEN in pediatric CD patients should be reconsidered before proceeding to a subsequent safety and efficacy trial. As described previously, EEN-induced mucosal healing is accompanied by changes in the gut microbiota [29,33]. Assuming that these microbial signatures directly mediate anti-inflammation and thereby clinical response, we hypothesized that EEN-conditioned stool could be used in an autologous FMT approach to preserve the beneficial effects of the nutritional therapy during the maintenance period. However, the gut microbial composition of CD patients has previously been found to paradoxically shift even more toward presumed dysbiosis during EEN, as evidenced by a further reduction of bacterial diversity [35,38,41], which directly correlates with treatment success [37,81,82]. Instead of observing a universal EEN microbiota composition shared between all patients, our results show a highly individual microbial composition under EEN therapy with great variation in Shannon-index and microbial richness. Accordingly, in nearly half of the fecal samples from our study cohort, microbial diversity and richness were particularly poor and below the minimal requirements of the FMT laboratory for normal allogenic FMT capsule production. Using such microbiota with low diversity communities for FMT may not seem promising, as studies on allogenic FMT in IBD identified a high donor microbial diversity as one of the most important factors of FMT success [14,83,84]. On the other hand, the metabolic changes occurring during EEN, including an improved bile acid metabolism and a reduction of potentially harmful microbial metabolites, correspond to a functional normalization of the gut microbiota [35]. Furthermore, as all patients were started on infliximab and methotrexate during EEN, most stool donations were collected under both EEN and maintenance therapy. Assumingly, such “double-conditioning” of the microbiota, e.g., EEN plus infliximab and methotrexate, might enhance the beneficial effects of autologous FMT. In a recent study, anti-TNF treatment was found to improve the previous low-diversity state by increasing gut microbial diversity and, e.g., normalizing the ratio of *Faecalibacterium prausnitzii* to *Escherichia coli* [85]. These findings suggest therapeutic effects of anti-TNF drugs go beyond simple anti-inflammation. However, such microbial changes were observed in stool samples collected 6 months after the initiation of anti-TNF treatment [85], which currently does not allow for decisive conclusions about short-term effects.

## 5. Conclusions

Based on a small case series of EEN-treated CD patients, we suspect that autologous FMT via capsules containing EEN-conditioned microbiota is unlikely to be a suitable therapeutic approach in pediatric CD. Given the emergence of reassuring safety data and promising results from recent trials in adult CD, allogeneic FMT appears to be a compelling alternative in pediatric patients, as well, and should be explored in further studies [9,10,15,16]. A multi-donor approach might be preferable as high microbial species richness and diversity have been linked to increased treatment success [14,83]. Concerning the recipient’s side, inflammation control should be achieved prior to FMT to alleviate immune pressure on the transferred microbiota, thereby facilitating its engraftment [10,11,12,13] and decreasing the probability of side effects [17]. In the long run, other live biotherapeutic products, such as naïve or engineered microbial consortiums and probiotics, could help solve the problem of donor selection and selectively correct individual microbial imbalances [66,86], which would represent an important step toward personalized medicine in IBD.

## Figures and Tables

**Table 1:** Demographic and clinical characteristics of the study population at baseline.

Pat.	Age at Diagnosis (Years)	Age at Study Inclusion (Years)	Newly Diagnosed	Symptoms at Presentation	Paris Classification *		Disease Activity (wPCDAI)
					Disease Location	Disease Behavior	
FMT-1	16.5	16.5	Yes	Diarrhea, reduced daily activity	L2, L4a	B1, G0	moderate (47.5)
FMT-2	15.2	15.2	Yes	Abdominal pain, diarrhea, involuntary weight loss	L4ab	B1, G0	moderate (45)
FMT-3	7.8	11.3	No	Bloody diarrhea, abdominal pain, vomiting, poor well-being, involuntary weight loss	L3, L4a	B1p, G1	severe (72.5)
FMT-4	9.2	15.3	No	Abdominal pain, diarrhea, poor well-being	L2, L4a	B1, G1	moderate (52.5)
FMT-5	12.8	12.8	Yes	Diarrhea, involuntary weight loss, anal abscess, uveitis	L3, L4ab	B3p, G0	moderate (42.5)
FMT-6	14.3	14.4	Yes	Abdominal pain	L1, L4a	B1, G1	mild (40)
FMT-7	11.3	11.3	Yes	Abdominal pain, vomiting, poor well-being, involuntary weight loss	L3, L4ab	B1, G0	severe (67.5)

* Paris classification [56].

**Table 2:** Test runs of FMT capsule production for potential autologous use.

Pat. *	EEN Weeks Completed at Time Point of Stool Donation	Stool Weight (g) from Single Donation	Bristol Stool Scale (1–7)	Bacterial Richness (Number of zOTUs at Genus Level Pre-/Post- Shipment)	Shannon-Index (Pre-/Post- Shipment)	Number of Capsules Produced ^1^
FMT-1	5	71	2	38/39	3.3/3.4	30
FMT-2	4	55	1	47/48	3.8/4.1	30
	5	201	2	48/49	4.0/3.9	30
FMT-3	3	68	6	18/16	2.6/2.4	30
	4	37	6	18/16	3.1/2.6	21
FMT-4	3	240	7	15/15	2.1/2.9	10
FMT-5	3	10	6	39/40	4.0/3.8	0
FMT-6	7	21	4	30/32	3.8/3.5	30
FMT-7	6	100	4	38/43	3.9/4.0	30

* Shown are characteristics of stool samples collected by study participants for potential autologous FMT. Patients FMT-2 and FMT-3 provided fecal material for two test runs. Fecal biomass and Bristol stool scale were assessed for each stool donation after transport to the FMT laboratory, followed by FMT donor safety screening and stool encapsulation. 16S rRNA gene sequencing was used to determine the bacterial richness (number of zOTUs at the genus level) and diversity (Shannon index) of each stool sample pre- and post-shipment to the FMT laboratory. ^1^ Whenever possible, a standard batch consisting of 30 capsules was produced. No capsules could be produced for patient FMT-5 as stool had leaked from the Fecotainer during transport. Abb.: zOTUs = zero-radius operational taxonomic units.

## Data Availability

Raw sequencing data were submitted to the European Nucleotide Archive (ENA) and are available under project accession number PRJEB55998. Further data underlying this article will be shared upon reasonable request to the corresponding author.

## Supplementary Materials

The following supporting information can be downloaded at: https://www.mdpi.com/article/10.3390/nu15071742/s1, Figure S1: Flow chart of the FMT capsule manufacturing process; Figure S2: Clinical course; Figure S3: Alpha-diversity analysis of stool samples from study participants pre- and post-shipment and after encapsulation; Figure S4: Beta diversity of fecal samples pre- and post-shipment and after encapsulation; Figure S5: Detection of the top 30 bacterial genera in stool samples pre- and post-shipment and after encapsulation; Figure S6: Relative bacterial counts of the mostly altered zOTUs post-shipment and after encapsulation; Figure S7: Absolute abundance of stool samples pre- and post-shipment and after encapsulation; Figure S8: Absolute bacterial counts of the mostly altered zOTUs pre- and post-shipment; Table S1: Stool pathogen screening; Table S2: Z-scores for height-for-age, weight-for-age, and BMI prior to and post-EEN; Table S3: Tests for significant differences in beta-diversity between sampling time points of stool processing.
