# Supplementary material for: Is Autologous Fecal Microbiota Transfer after Exclusive Enteral Nutrition in Pediatric Crohn’s Disease Patients Rational and Feasible? Data from a Feasibility Test
Source: Nutrients. 2023 Apr 2;15(7):1742. doi: 10.3390/nu15071742 (PMC10096730; doi:10.3390/nu15071742)
Supplement: Supplementary file 1 [file nutrients-15-01742-s001.zip › nutrients-2254431-supplementary.pdf]

nutrients

Article

Is Autologous Fecal Microbiota Transfer after Exclusive Enteral Nutrition in Pediatric Crohn's Disease Patients Rational and Feasible? Data from a Feasibility Test

Hannes Hoelz [1], Jeannine Heetmeyer [1], Anastasia Tsakmaklis [2], Andreas Hiergeist [3], Kolja Siebert [1], Federica De Zen [1], Deborah Häcker [4], Amira Metwaly [4], Klaus Neuhaus [5], André Gessner [3], Maria J. G. T. Vehreschild [2,6], Dirk Haller [4,5] and Tobias Schwerd [1,*]

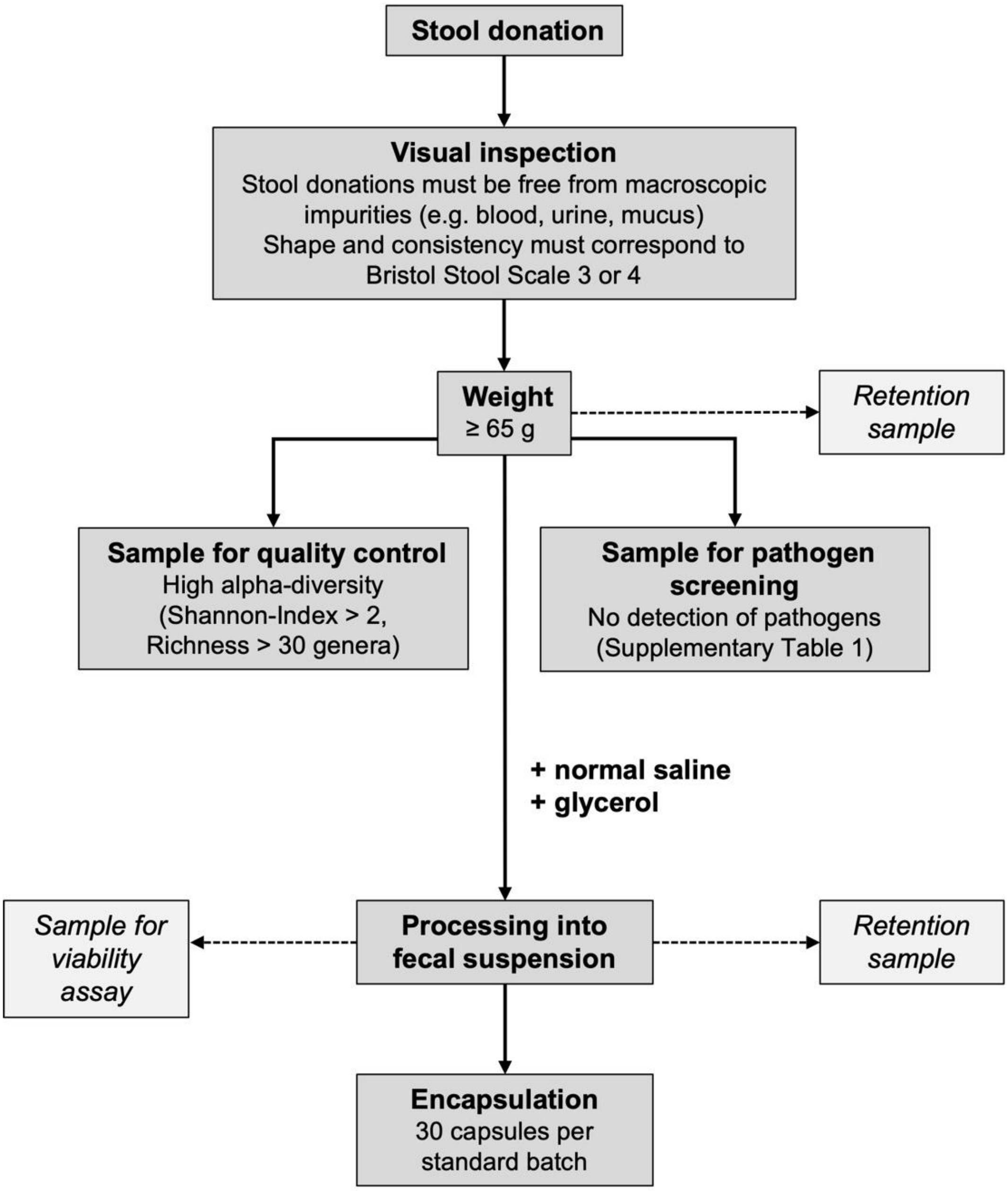

Figure S1. Flow chart of the FMT capsule manufacturing process.

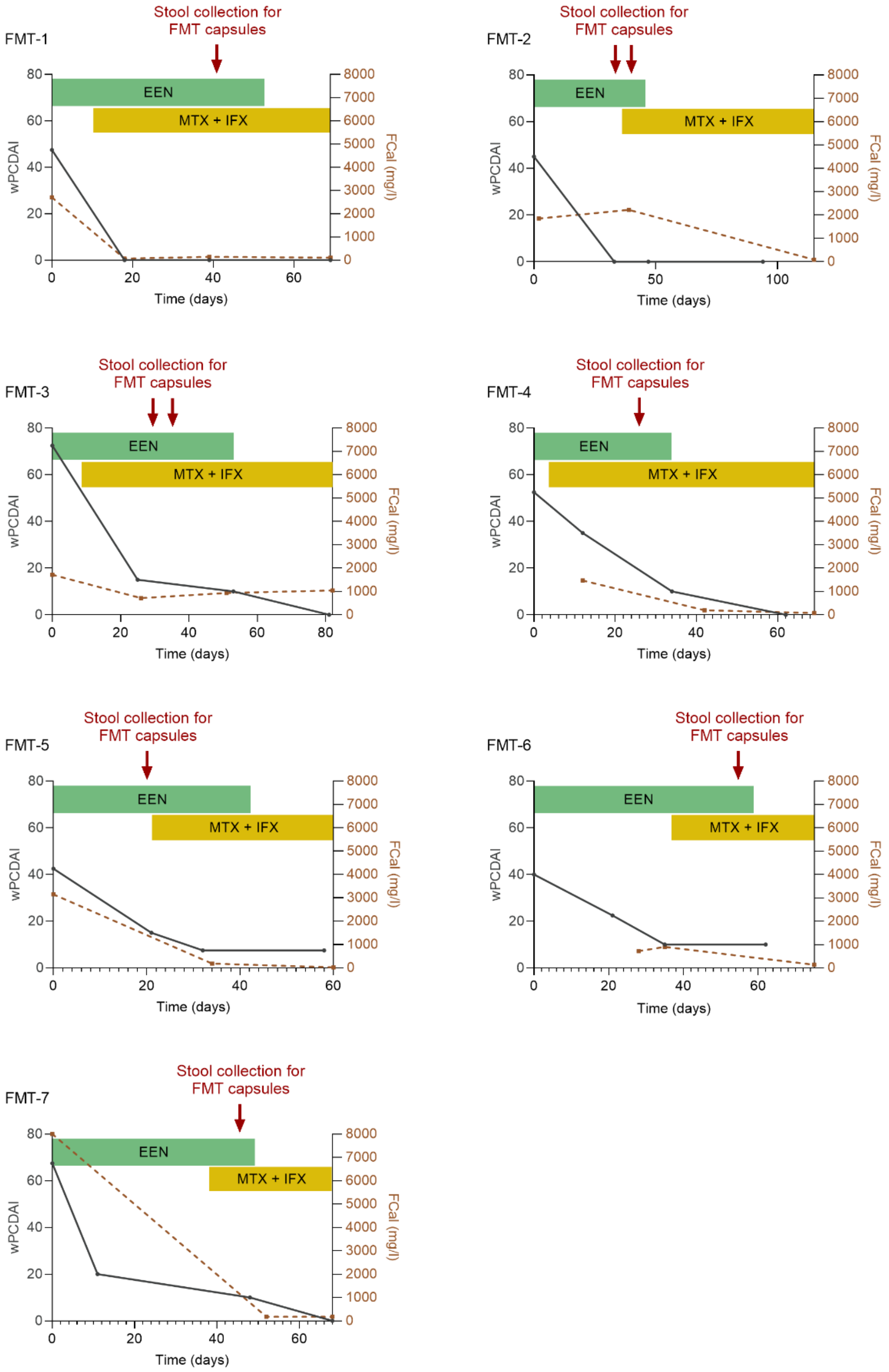

Figure S2. Clinical course**.** Clinical course of each study participant including wPCDAI scores, fCal concentrations and received treatments over a period of max. 115 days. Green: Induction therapy with EEN. Ocher: Maintenance therapy with IFX and MTX. Red arrow: Time point of stool collection.

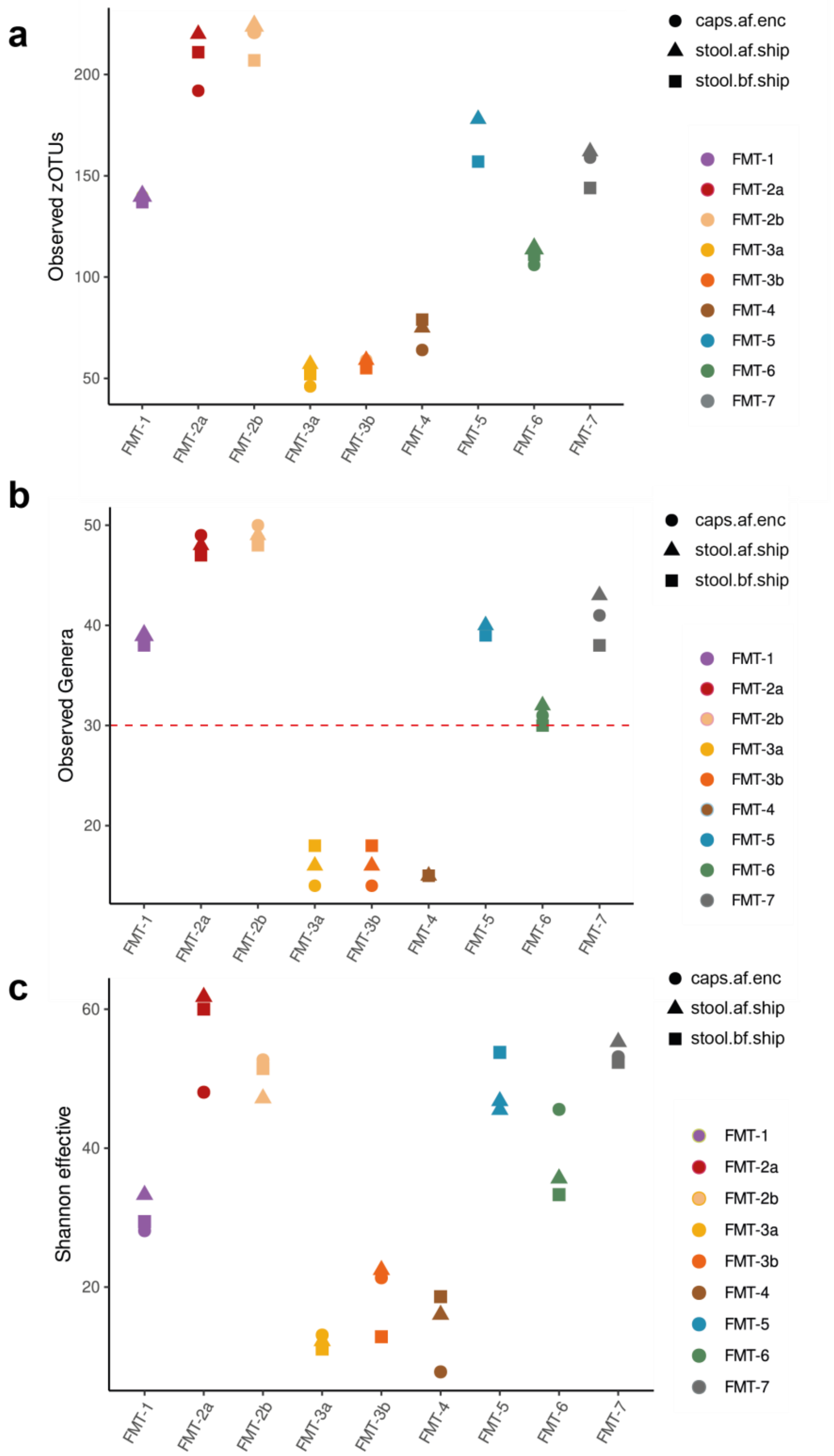

Figure S3. Alpha-diversity analysis of stool samples from study participants pre- and post-shipment and after encapsulation. Alpha-diversity analysis measured by **(a)** the number of observed zOTUs, **(b)** the number of observed genera and **(c)** the Shannon effective (number of species) in stool samples from study participants pre- and post-shipment and after encapsulation as determined by 16S rRNA sequencing. Dotted red line in **(b)** indicates minimum number of genera (richness) required for a FMT product for use in humans according to the quality criteria for high alpha-diversity in the FMT laboratory in Cologne.

Note: Patients FMT-2 and FMT-3 provided fecal material for two test runs (FMT-2a/b and FMT-3a/b). Two samples taken post-transport were analyzed for patient FMT-5 and no capsules could be produced from the fecal material donated by this patient due to stool leakage from the Fecotainer during transport.

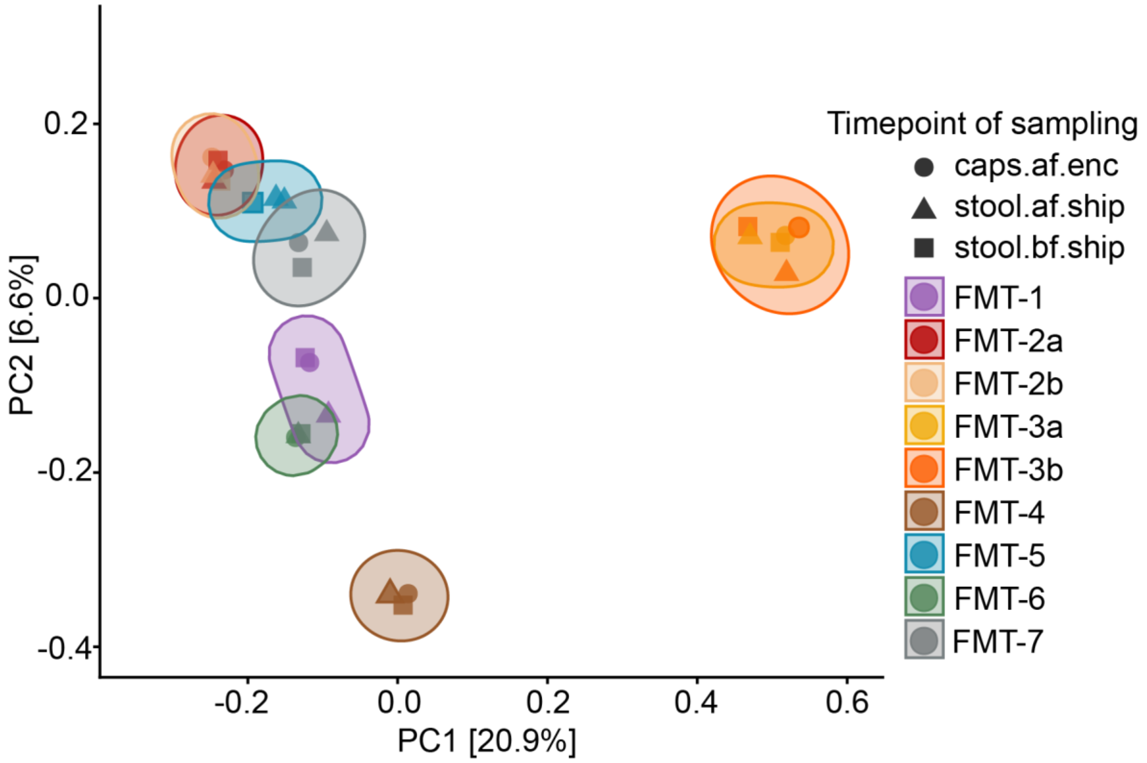

Figure S4. Beta diversity of fecal samples pre- and post-shipment and after encapsulation. Beta diversity measured by unweighted UniFrac dissimilarity and principal coordinates analysis (PCoA) plotted for fecal samples pre- (■) and post-shipment (▲) and after encapsulation (●). A PERMANOVA-test was performed to test for significance (confidence level 95%, definition of statistical significance: $p < 0.05$). Linear models were fitted to distance matrices using the adonis2 command in the vegan package [1]. Patients were included as covariate into the model to control for individual differences. Results were considered significant for $p \leq 0.05$. For pairwise comparisons, p-values were adjusted by false discovery rate using the Benjamini-Hochberg procedure. A pairwise comparison of stool before and after shipment (p=0.12), stool before shipment and after encapsulation (p=0.10) and stool after shipment and after encapsulation (p=0.06) showed no statistically significant difference, respectively.

Note: Patients FMT-2 and FMT-3 provided fecal material for two test runs (FMT-2a/b and FMT-3a/b). Two samples taken post-transport were analyzed for patient FMT-5 and no capsules could be produced from the fecal material donated by this patient due to stool leakage from the Fecotainer during transport.

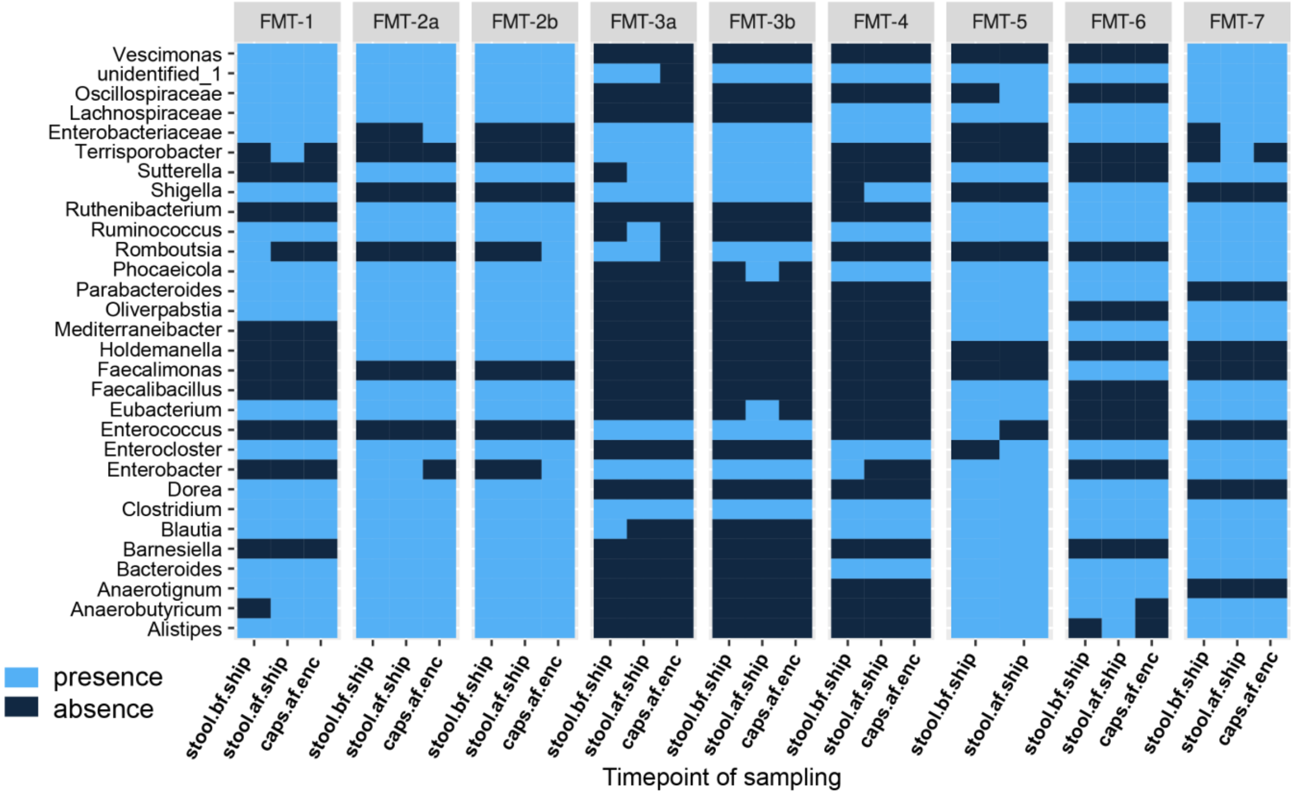

Figure S5. Detection of the top 30 bacterial genera in stool samples pre- and post-shipment and after encapsulation. Heatmap showing the presence or absence of the top 30 bacterial genera in stool samples from study participants pre- (stool.bf.ship) and post-shipment (stool.af.ship) and after encapsulation (caps.af.enc) as determined by 16S rRNA sequencing. **Note:** Patients FMT-2 and FMT-3 provided fecal material for two test runs (FMT-2a/b and FMT-3a/b). Two samples taken post-transport were analyzed for patient FMT-5 and no capsules could be produced from the fecal material donated by this patient due to stool leakage from the Fecotainer during transport.

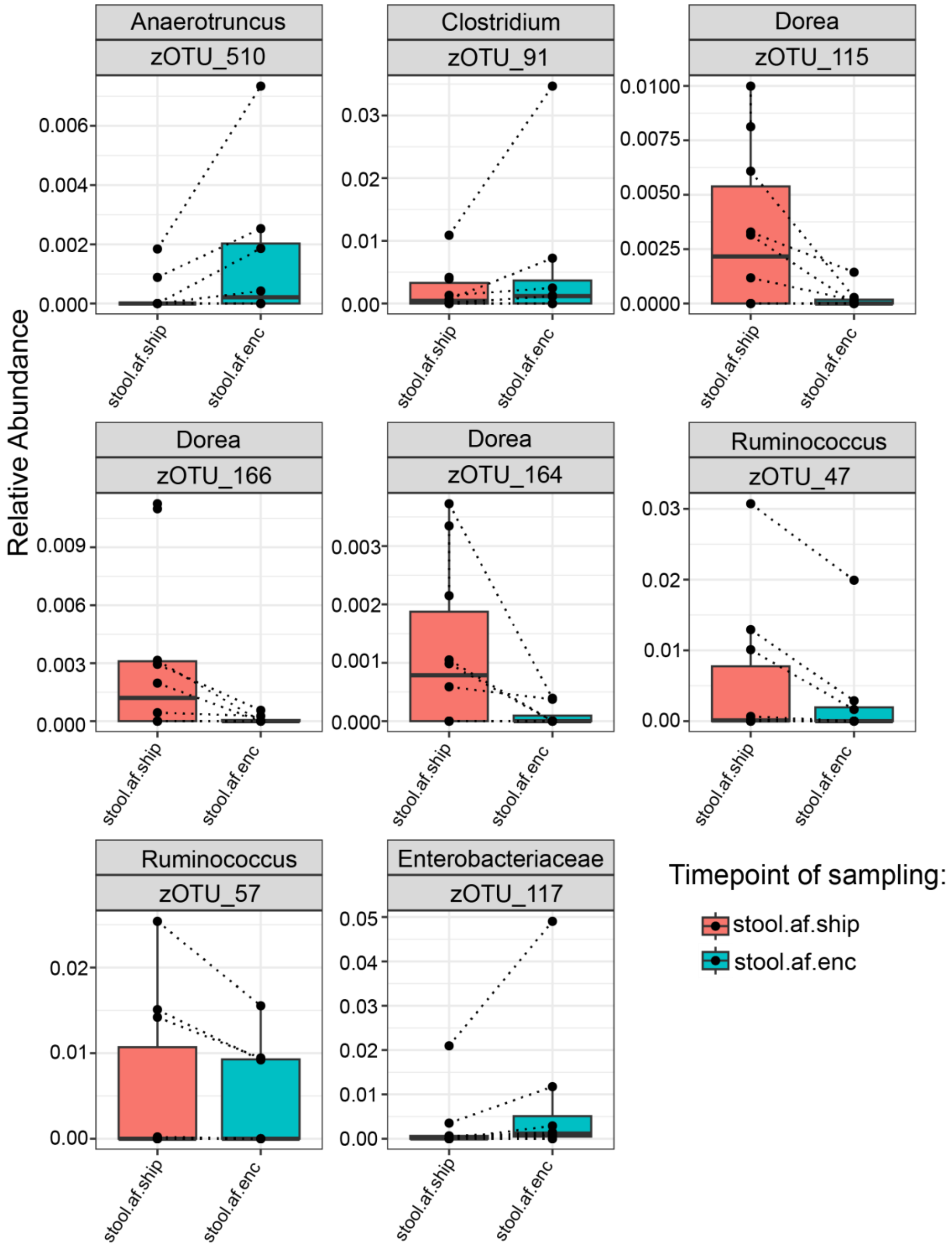

Figure S6. Relative bacterial counts of the mostly altered zOTUs post-shipment and after encapsulation**.** Boxplots of relative bacterial counts (zOTUs) post-shipment (stool.af.ship) and after encapsulation (stool.af.enc) based on bacterial abundances of zOTUs. To identify significantly different zOTUs after shipment and after encapsulation the MaAsLin2 package in R, a TSS-normalized, log-transformed linear model for differential abundance analyses, was used [2]. Timepoint of sampling was included as fixed effects and the individual patiens as random effects into the model. Correction for multiple testing was performed using the Benjamini-Hochberg FDR threshold of 0.25. A cutoff value of 0.05 was applied for the uncorrected p-value to plot relative abudances of each group. The zOTUs with the greatest shifts of relative zOTU counts are depicted, but none of them was statistically significant.

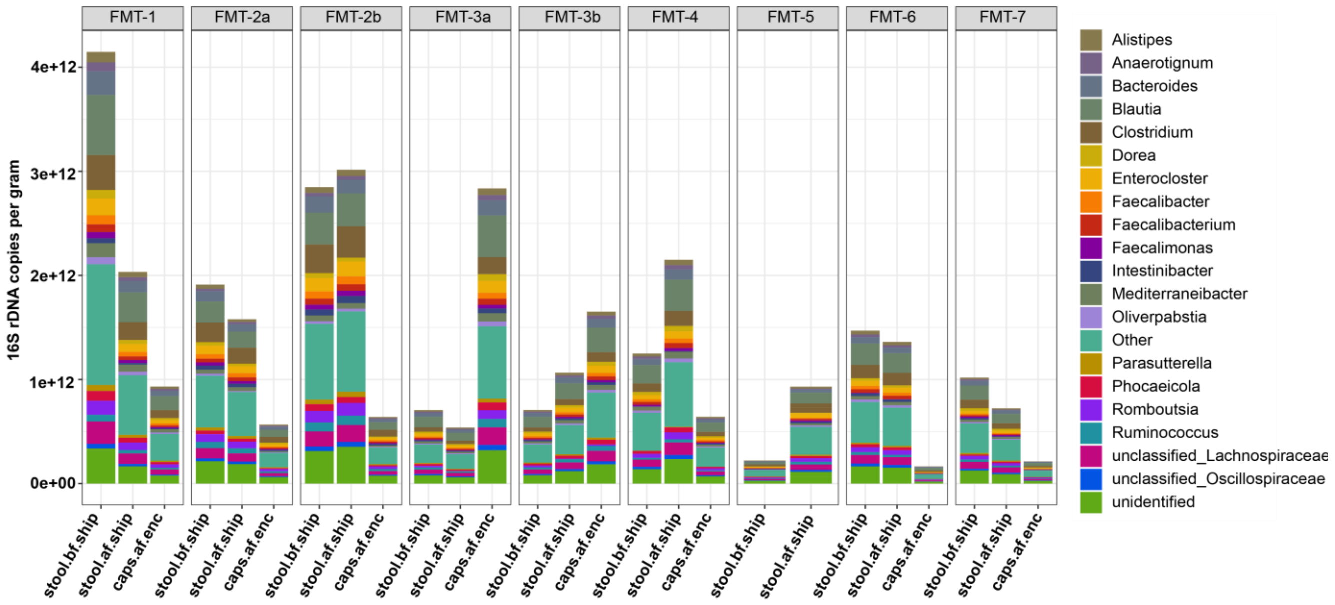

Figure S7. Absolute abundance of stool samples pre- and post shipment and after encapsulation**.** Stacked bar charts of absolute bacterial counts pre- (stool.bf.ship) and post-shipment (stool.af.ship) as well as after encapsulation (caps.af.enc) based on bacterial abundances at the family level normalized to 16S rDNA copies determined by qRT-PCR.

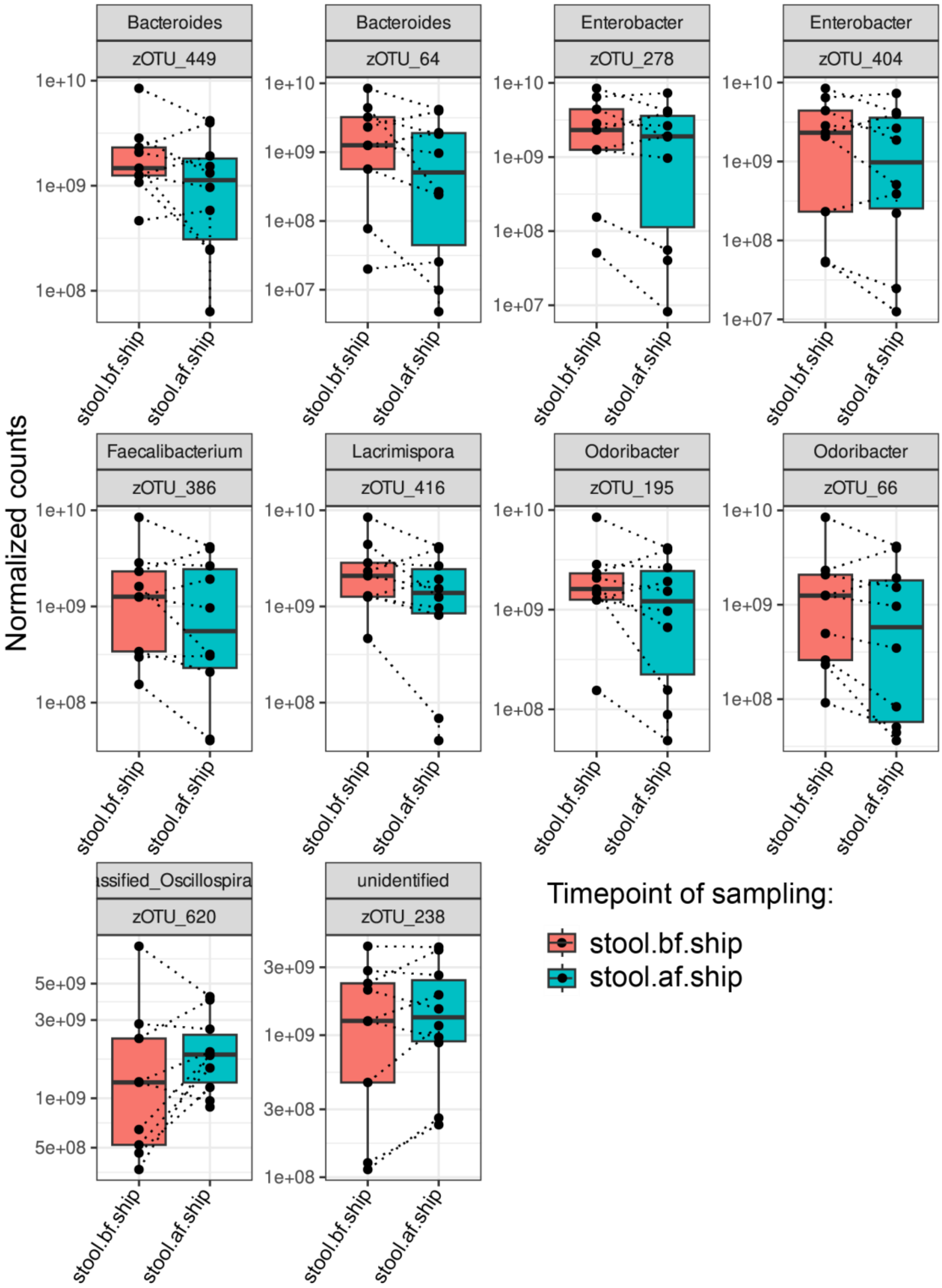

Figure S8. Absolute bacterial counts of the mostly altered zOTUs pre- and post-shipment. Boxplots of absolute bacterial counts (zOTUs) pre- (stool.bf.ship) and post-shipment (stool.af.ship) based on bacterial abundances of zOTUs normalized to 16S rRNA copies. To identify significantly different zOTUs before and after shipment the MaAsLin2 package in R, a TSS-normalized, log-transformed linear model for differential abundance analyses, was used [2]. Timepoint of sampling was included as fixed effects and the individual patiens as random effects into the model. Correction for multiple testing was performed using the Benjamini-Hochberg FDR threshold of 0.25. A cutoff value of 0.05 was applied for the uncorrected p-value to plot abundances of each group. The 10 zOTUs with the greatest shifts of normalized zOTU counts are depicted. Though small shifts of normalized zOTU counts occurred, no significant changes of zOTUs were observed. The analysis showed no evidence of a systematic influence of a cooled Fecotainer transport at 4°C on the occurrence of these zOTUs.

Table S1. Stool pathogen screening.

**Culture**			
Bacteria			Fungi
Listeria monocytogenes*			Candida auris*
Methicillin-resistant Staphylococcus aureus (MRSA)*			Candida albicans
Vancomycin-resistant Enterococci (VRE)*			
3/4 MDRGN, including extended-spectrum β-lactamase (ESBL)-producing Enterobacteriaceae*			
**PCR**			
Bacteria	Antibiotic resistance genes	Viruses	Parasites
Salmonella spp.*	Extended-spectrum β-lactamases (ESBL)*	Norovirus GI/GII*	Entamoeba histolytica*
Shigella*	Imipenemase (IMP)-type metallo-β-lactamase	Adenovirus F40/41*	Giardia lamblia*
Vibrio (parahaemolyticus, vulnificus, and cholerae)*	Klebsiella pneumoniae carbapenemase (KPC)	Astrovirus*	Cryptosporidium spp.*
Vibrio cholerae*	New Delhi metallo-β-lactamase (NDM)	Enterovirus*	Cyclospora cayetanensis*
Campylobacter (jejuni, coli, and upsaliensis)*	Oxacillinase-48 (OXA-48)	Rotavirus A*	Blastocystis hominis*
Yersinia enterocolitica*	Vancomycin-resistant Enterococci (vanA/vanB)	Sapovirus (I, II, IV, and V)*	Dientamoeba fragilis*
Clostridioides difficile Toxin A and B*	Verona integron-encoded metallo-β-lactamase (VIM)	SARS-CoV-2	Cystoisospora belli*
Methicillin-resistant Staphylococcus aureus (MRSA)*		Parechovirus	Microsporidia (Enterocytozoon spp./ Encephalitozon spp.)*
E. coli O157 (EHEC)*			Enterobius vermicularis
Shiga-like toxin-producing E. coli (STEC) stx1/stx2*			Strongyloides spp.
EAEC, EPEC, ETEC, EIEC*			Hymenolepis spp.
Plesiomonas shigelloides*			Necator americanus
Tropheryma whipplei			Taenia spp.
Aeromonas spp.			Trichuris trichiura
**Antigen Test**			
Helicobacter pylori*			
**Microscopy**			
Ova, cysts, parasites*			

* Testing required by BfArM for FMT donor screening. Abb.: EAEC = enteroaggregative Escherichia coli, EIEC = enteroinvasive Escherichia coli, EHEC = enterohemorrhagic E. coli, EPEC = enteropathogenic E. coli, ETEC = enterotoxigenic E. coli, MDRGN = multidrug-resistant Gram-negative bacteria, spp. = species, SARS-CoV-2 = severe acute respiratory syndrome coronavirus type 2.

Table S2. Z-scores for height-for-age, weight-for-age and BMI prior to and post EEN.

**Pat.**	**Pre-EEN**			**Post-EEN**		
	**Height**	**Weight**	**BMI**	**Height**	**Weight**	**BMI***
FMT-1	0.96	1.77	1.58	1.11	1.75	1.52
FMT-2	0.39	−0.82	−1.76	0.87	0.14	−0.52
FMT-3	−0.81	−0.89	−0.44	−0.55	−0.26	0.16
FMT-4	−0.38	0.13	0.29	−0.01	−0.11	−0.27
FMT-5	0.59	−0.54	−1.24	0.54	0.3	0.13
FMT-6	−1.55	−2.7	**−2.32**	−1.44	−2.17	−1.74
FMT-7	1.69	0.49	−0.26	1.72	1.05	0.6

* z-scores were calculated using the following anthropometric database as reference: Centers for Disease Control and Prevention (CDC), National Center for Health Statistics. CDC growth charts: United States. http://www.cdc.gov/growthcharts/. May 30, 2000. Severe malnourishment was defined according to World Health Organization (WHO) as a BMI z-score <-2. No statistical difference between pre- and post-EEN BMI z-scores was observed (p=0.069, paired t-test).

Table S3. Tests for significant differences in beta-diversity between sampling time points of stool processing.

**Pairwise comparison of sampling time points of stool processing***	**Pre- vs. post-shipment**	**Pre shipment vs. encapsulation**	**Post shipment vs. encapsulation**
Bray-Curtis	0.10	0.18	**0.03**
Weighted UniFrac	0.41	0.07	**0.04**
Unweighted UniFrac	0.14	0.10	0.06
Generalized UniFrac	0.09	0.16	**0.02**

* Depicted are p-values from pairwise comparison based on Bray-Curtis, unweighted and weighted, as well as generalized Unifrac distances using PERMANOVA analysis. Significant p-values are highlighted with bold numbers. Linear models were fitted to distance matrices using the adonis2 command in the vegan package [1]. Patients were included as covariate into the model to control for individual differences. Results were considered significant for p ≤ 0.05. For pairwise comparisons, p-values were adjusted by false discovery rate using the Benjamini-Hochberg procedure.

References

1. Dixon, P. VEGAN, a Package of R Functions for Community Ecology. *J. Veg. Sci.* **2003**, *14*, 927–930, doi:10.1111/j.1654-1103.2003.tb02228.x.
2. Mallick, H.; Rahnavard, A.; McIver, L.J.; Ma, S.; Zhang, Y.; Nguyen, L.H.; Tickle, T.L.; Weingart, G.; Ren, B.; Schwager, E.H.; et al. Multivariable Association Discovery in Population-Scale Meta-Omics Studies. *PLoS Comput. Biol.* **2021**, *17*, e1009442, doi:10.1371/journal.pcbi.1009442.
